# Filamentous surface structures drive biofilm formation in ICU-isolated *Acinetobacter baumannii*, *Pseudomonas aeruginosa*, and *Staphylococcus aureus*: implications for persistent environmental contamination

**DOI:** 10.1128/spectrum.02114-25

**Published:** 2026-03-13

**Authors:** Bing Wang, Kaiwen Ni, Weiran Wang, Min Lu, Hui Jin

**Affiliations:** 1 Department of Disinfection Surveillance and Vector Control, Hangzhou Center of Disease Control and Prevention (Hangzhou Health Supervision Institution), Hangzhou, Zhejiang, China; 2 Zhejiang Key Laboratory of Multi-Omics in Infection and Immunity, Hangzhou, Zhejiang, China; 3 Department of Infection Control, Second Affiliated Hospital of Zhejiang University School of Medicine, Hangzhou, Zhejiang, China; UCI Health, Orange, California, USA

**Keywords:** intensive care unit, biofilm formation, *Acinetobacter baumannii*, *Pseudomonas aeruginosa*, *Staphylococcus aureus*

## Abstract

The relationship between surface biofilms and the persistent contamination of environmental surfaces has garnered increasing attention. Although biofilm research is extensive, systematic comparisons of the morphological characteristics of pathogens isolated from healthcare surfaces, particularly strains that retain biofilm-forming capacity after long-term preservation, are still lacking. In this study, we aimed to investigate the biofilm formation potential and ultrastructural features of the cell membrane surfaces of healthcare environment-isolated *Acinetobacter baumannii*, *Pseudomonas aeruginosa*, and *Staphylococcus aureus*. The revived strains were inoculated into 96-well plates containing TSB and incubated for 24 h to assess their biofilm-forming potential under favorable growth conditions. Biofilm formation was assessed using crystal violet staining. Strains with different biofilm-forming abilities were examined for their morphological characteristics under a scanning electron microscope. Of the 198 historically preserved strains of the three common pathogens, 162 were successfully revived (78 *A. baumannii*, 36 *P. aeruginosa*, and 48 *S. aureus*). The biofilm formation rates of *A. baumannii*, *P. aeruginosa*, and *S. aureus* were 70.51%, 88.89%, and 25%, respectively. The cell surface morphology between the biofilm- and non-biofilm-forming strains differed significantly. Biofilm-forming strains exhibited numerous filamentous structures on their surfaces and displayed aggregation and multidimensional stacking owing to the “net-like” effect of the filaments. In contrast, non-biofilm-forming strains had smooth surfaces without filamentous structures or aggregation. This study provides systematic evidence for the biofilm-forming capabilities of common pathogens associated with healthcare-associated infections, isolated from healthcare environment surfaces. All biofilm-forming strains displayed distinct filamentous structures on their surfaces, with the clonal strains exhibiting similar characteristics.

**IMPORTANCE:**

This study is the first to demonstrate the biofilm-forming capacity and morphological characteristics of *Acinetobacter baumannii*, *Pseudomonas aeruginosa*, and *Staphylococcus aureus* in intensive care unit (ICU) environments, filling a critical gap in our understanding of biofilm mechanisms among healthcare-associated pathogens. Notably, *P. aeruginosa* exhibited an 88.89% biofilm formation rate, with its distinctive filamentous fibrous structures significantly enhancing bacterial adhesion and aggregation—a key explanation for persistent environmental contamination. These findings directly inform the optimization of ICU cleaning protocols, promote the development of biofilm-targeted disinfection standards, and provide a scientific foundation for refining environmental monitoring metrics in infection control policies, ultimately reducing healthcare-associated infection rates.

## INTRODUCTION

Healthcare-associated infections (HAIs) pose the greatest challenges to patient safety globally (1, 2). Recently, the role of hospital environmental hygiene (HEH) in infection prevention and control (IPC) was reevaluated. Strengthening HEH improves patient safety and reduces the incidence of HAIs and/or the colonization rates of pathogenic microorganisms (3–5), while delivering significant cost benefits, such as averting extra treatment costs due to infections, improving patient outcomes, reducing mortality and suffering, and enhancing the overall operational efficiency of the healthcare system (2, 6).

Pathogenic bacteria responsible for environmental contamination in healthcare facilities can adhere to surfaces and form biofilms that are difficult to eradicate (7). Thus, these biofilms serve as persistent sources of contamination in hospital environments (8, 9). More than 65% of HAIs have been estimated to be directly associated with biofilm formation (10). In 2012, Vickery et al. first described bacterial biofilm formation by methicillin-resistant *Staphylococcus aureus* on dry surfaces such as bedding, curtains, and furniture, in intensive care units (ICUs) (11). Subsequently, Hu et al. suggested that HAI-causing pathogens originate from non-living environmental surfaces surrounding patients, attributing the persistence of these biofilms to the protective role of dry surface biofilms (DSBs) (9).

Biofilms are ubiquitous in healthcare settings and can be classified into two distinct types based on their hydration state and formation pathway. Hydrated biofilms, commonly found in waterway pipes and lumen-type medical devices, form in moistened environments where surfaces are hydrated through various means. In contrast, DSBs on non-living environmental and medical equipment surfaces (8, 9, 12) arise primarily from the drying of cells on surfaces, rather than from *de novo* metabolic construction under dry conditions. Research on biofilms has been a focal point in healthcare as the ability of bacteria to form biofilms is strongly associated with clinical issues such as chronic wounds, urinary tract infections, pneumonia, and device-related infections (13–15). The diffusion and transmission of pathogens from biofilms, whether within patients with invasive device-related infections or on frequently touched medical equipment, can represent major sources of patient infections, thereby posing a substantial infection risk (16). However, research on DSBs is lagging behind that of hydrated biofilms, with only a limited number of groundbreaking advances. A recent systematic review highlighted that current infection control research primarily focuses on improving environmental cleaning strategies, training, and feedback from environmental service workers and introducing new disinfection technologies (17). In contrast, studies on DSBs are relatively scarce, and there is a lack of clinical frontline data on the biofilm-forming capabilities of HAI pathogens on surfaces in healthcare environments.

This study focused on historical samples preserved in our laboratory from environmental surfaces of healthcare facilities, specifically *Acinetobacter baumannii*, *Pseudomonas aeruginosa*, and *S. aureus*. We aimed to address the following key points: first, to confirm whether these three common HAI pathogens universally possess the ability to produce biofilms; second, to analyze the differences in biofilm-formation capabilities among the three pathogens; and third, to observe the morphological characteristics of bacterial cell membranes during the early stages of biofilm formation (24 h). Our findings will provide a scientific basis for further exploration of the mechanisms of DSB formation and strategies for IPC.

## RESULTS

### Detection and differential analysis of biofilm formation ability

In total, 198 strains were isolated from the high-touch surfaces of six ICUs. We successfully revived 162 strains, resulting in a recovery rate of 81.82%. The remaining strains failed to grow under the standard revival conditions, which may be attributed to cumulative cryodamage or loss of viability during long-term storage. There were 78 *A. baumannii*, 36 *P. aeruginosa*, and 48 *S. aureus* strains. The results (Table 1) indicated that among *A. baumannii* strains, 70.51% were capable of biofilm formation, with 22 strains (28.21%) classified as strong biofilm-forming (SB), 23 strains (29.49%) as moderate biofilm-forming (MB), 10 strains (12.82%) as weak biofilm-forming (WB), and 23 strains as non-biofilm forming (NB). Among *P. aeruginosa* strains, 88.89% produced biofilms; among these, 5 strains (13.89%) were classified as SB, 27 strains (75.00%) as MB, and 4 strains as NB. Among *S. aureus* strains, 25.00% formed biofilms, with 3 strains (6.25%) classified as MB, 9 strains (18.75%) as WB, and 36 strains as NB. Raw OD data for all strains, including classification cutoffs, are provided in Fig. S1 to S3. The distribution of biofilm formation capacity differed significantly among *A. baumannii*, *P. aeruginosa*, and *S. aureus* (Pearson’s *χ*² test, *P* < 0.001).

**TABLE 1:** Biofilm-forming ability of different strains and statistical comparisons^*a*^

Strain	NB	WB	MB	SB	Biofilm formation rate (%)
*Acinetobacter baumannii*	23 (29.49%)	10 (12.82%)	23 (29.49%)	22 (28.21%)	70.51
*Pseudomonas aeruginosa*	4 (11.11%)	0 (0.00%)	27 (75.00%)	5 (13.89%)	88.89
*Staphylococcus aureus*	36 (75.00%)	9 (18.75%)	3 (6.25%)	0 (0.00%)	25.00
Total	63	19	53	27	61.11

^*a*^ Overall Pearson’s *χ*^2^ test, *P* < 0.001. Post-hoc pairwise comparisons (with Bonferroni correction, *α*′ = 0.017): *A. baumannii* versus *P. aeruginosa*: *χ*^2^ = 8.15, *P* = 0.043 (ns). *A. baumannii* versus *S. aureus*: *χ*^2^ = 25.63, *P* < 0.001*. *P. aeruginosa* versus *S. aureus*: χ^2^ = 40.12, *P* < 0.001*. MB: moderate biofilm-forming; NB: non-biofilm-forming; ns: not significant after adjustment; SB: strong biofilm-forming; WB: weak biofilm-forming; *: statistically significant after adjustment.

### Microscopic features of cell membrane surfaces

Field-emission scanning electron microscopy (FE-SEM) showed significant differences in the cell surface structures between biofilm-forming and NB strains. These differences provide important evidence for understanding the biofilm-forming ability of bacteria.

**Fig 1:**
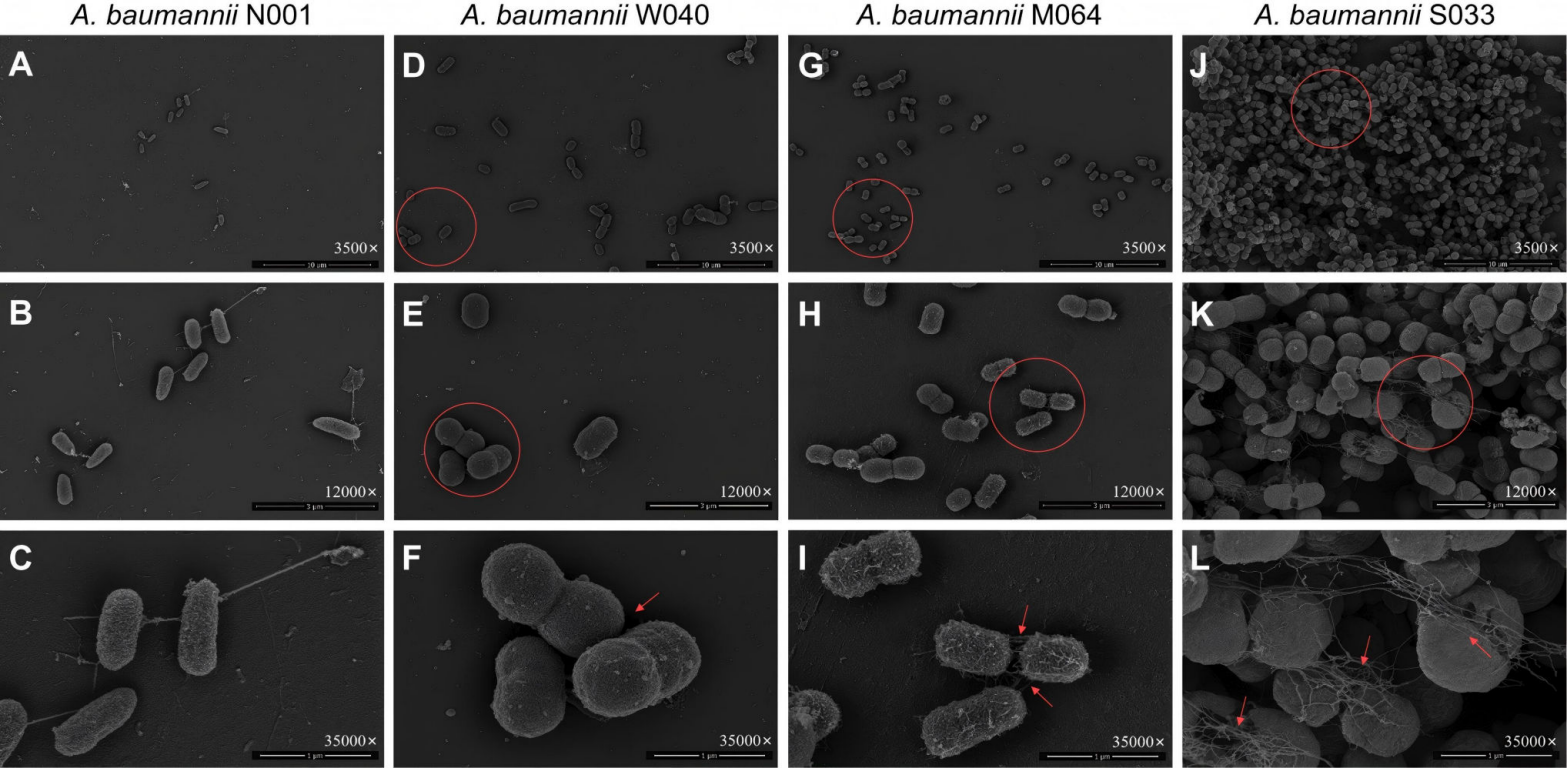
Morphological characteristics of *Acinetobacter baumannii* observed using SEM. Representative images are shown. For each strain, at least three random and non-contiguous fields of view were examined; the presented images illustrate the morphological features most consistently observed across these replicates. (**A–C**) NB strain N001 (sparse bacterial distribution with smooth cell surface). (**D–F**) WB strain W040 (cell aggregation and no apparent filamentous fibers). (**G–I**) MB strain M064 (cell aggregation and emerging short filamentous fibers). (**J–L**) SB strains S033 (dense cell aggregation and short fibers entangling cells). MB strain, moderate biofilm-forming strain; NB strain, non-biofilm-forming strain; SB strain, strong biofilm-forming strain; WB strain, weak biofilm-forming strain. (a/d/g/j) 3,500×, scale bar = 10 µm. (b/e/h/k) 12,000×, scale bar = 3 µm. (c/f/i/l) 35,000×, scale bar = 1 µm.

Figure 1 illustrates the morphological characteristics of *A. baumannii* strains observed using electron microscopy. The NB strain (N001) exhibited a relatively smooth cell surface, with a dispersed distribution of bacteria (Fig. 1A through C). The WB strain (W040) showed a rougher cell surface, but the bacteria remained dispersed with no aggregation (Fig. 1D through F). The MB strain (M054) displayed short filamentous fibers on the cell surface and exhibited a trend toward cell aggregation. At a magnification of 35,000×, filamentous fibers were clearly observed connecting the clonal strains (Fig. 1G through I). In the SB strain (S033), even at 3,500×, cells aggregated and overlapped owing to the “net-like” accumulation of surface filamentous fibers; this was even more pronounced at 12,000×. These fibers tightly connected adjacent cells and extended across multiple cell bodies, linking various cells together. Some fibers intertwined to form thick bundles, trapping a large number of cells within (Fig. 1J through L).

**Fig 2:**
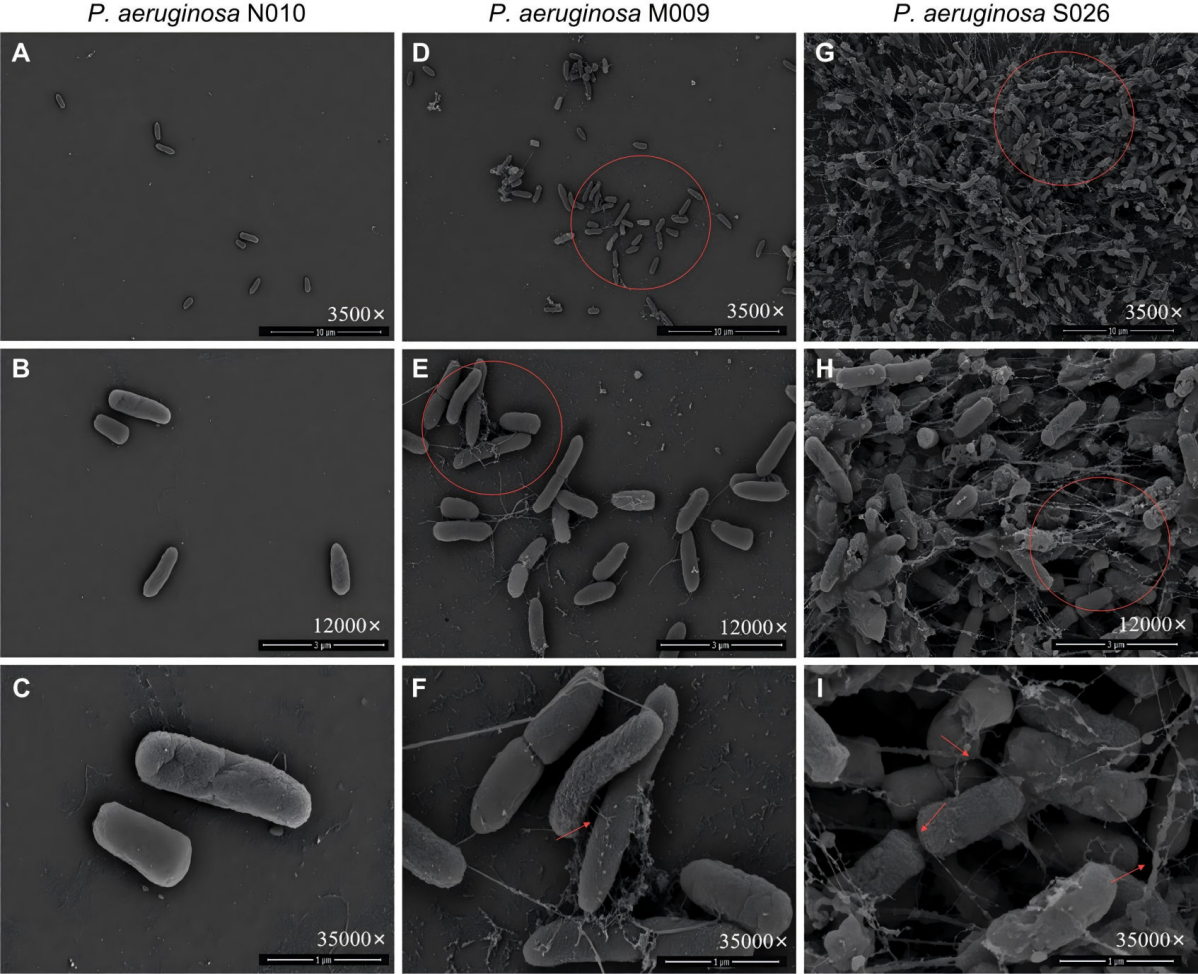
Morphological characteristics of *Pseudomonas aeruginosa* observed using SEM. Representative images are shown. For each strain, at least three random and non-contiguous fields of view were examined; the presented images illustrate the morphological features most consistently observed across these replicates. (**A–C**) NB strain N010 (sparse bacterial distribution with smooth cell surface). (**D–F**) MB strain M009 (cell aggregation and emerging short filamentous fibers). (**G–I**) SB strain S026 (dense cell aggregation and short fibers entangling cells). MB strain, moderate biofilm-forming strain; NB strain, non-biofilm-forming strain; SB strain, strong biofilm-forming strain; WB strain, weak biofilm-forming strain. (a/d/g) 3,500×, scale bar = 10 µm. (b/e/h) 12,000×, scale bar = 3 µm. (c/f/i) 35,000×, scale bar = 1 µm.

Figure 2 shows the morphological characteristics of *P. aeruginosa*. The NB strain (N010) appeared sparse on electron microscopy, with a smooth and flat cell surface and dispersed distribution (Fig. 2A through C). No WB strains were detected; the MB strain (M009) displayed long filamentous fibers that were intricately interconnected on the cell surface, along with noticeable cell aggregation and overlapping phenomena (Fig. 2D through F). The SB strain (S026) exhibited even complex and dense filamentous fiber connections, with a large number of cells clustered together, resulting in a distinct multidimensional stacking phenomenon (Fig. 2G through I).

**Fig 3:**
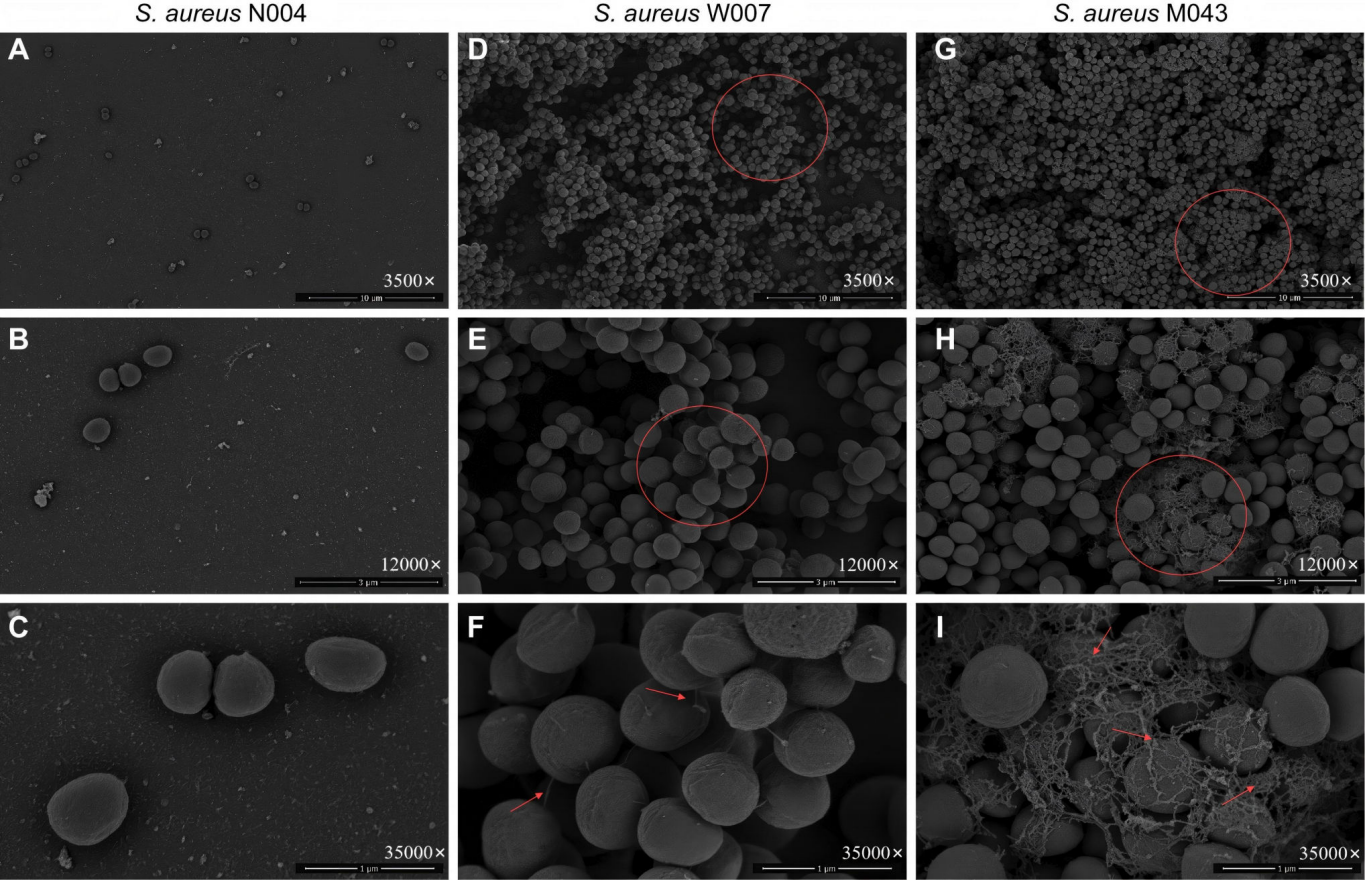
Morphological characteristics of *Staphylococcus aureus* observed using SEM. Representative images are shown. For each strain, at least three random and non-contiguous fields of view were examined; the presented images illustrate the morphological features most consistently observed across these replicates. (**A–C**) NB strain N004 (sparse bacterial distribution with smooth cell surface). (**D–F**) WB strain W007 (cell aggregation and no apparent filamentous fibers). (**G–I**) MB strain M043 (cell aggregation and emerging short filamentous fibers, some fibers trapping a large number of cells within). MB strain, moderate biofilm-forming strain; NB strain, non-biofilm-forming strain; SB strain, strong biofilm-forming strain; WB strain, weak biofilm-forming strain. (a/d/g) 3,500×, scale bar = 10 µm. (b/e/h) 12,000×, scale bar = 3 µm. (c/f/i) 35,000×, scale bar = 1 µm.

Figure 3 shows the morphological characteristics of *S. aureus*. The NB strain (N004) appeared sparse on electron microscopy, with a relatively smooth and flat cell surface at a high magnification (35,000×) (Fig. 3A through C). The WB strain (8) displayed a rougher cell surface than the NB strain, showing signs of cell aggregation, but no notable filamentous materials were observed on the cell surface (Fig. 3D through F). The MB strain (M043) exhibited aggregation on electron microscopy and, at a high magnification (35,000×), numerous cells were observed to be “ensnared” by filamentous fibers. However, the filamentous fibers on its surface were shorter, yet denser, than those of the aforementioned two gram-negative bacteria (Fig. 3G through I). No SB strains of *S. aureus* were detected.

## DISCUSSION

This study provides the evidence that biofilm-forming strains are prevalent among populations of *A. baumannii*, *P. aeruginosa*, and *S. aureus* isolated from ICU environmental surfaces. Statistical analysis confirmed marked differences in biofilm-forming propensity among the three species (*P* < 0.001). Post-hoc comparisons indicated that *P. aeruginosa* exhibited a significantly higher biofilm formation rate than *S. aureus*, and *A. baumannii* also formed biofilms more readily than *S. aureus* (both *P* < 0.001 after correction). Although the rate for *P. aeruginosa* was numerically higher than that of *A. baumannii*, this difference did not reach statistical significance following strict adjustment for multiple comparisons. The notably high biofilm formation rate of *P. aeruginosa* (88.89%) suggests a superior capacity for initial attachment and aggregation, which may be a key driver of its role as a persistent environmental contaminant in ICUs. The biofilm formation rates for *A. baumannii* and *S. aureus* were 70.51% and 25.00%, respectively. Notably, despite being preserved for approximately 9–12 years, these bacterial strains retained their biofilm-forming ability. Long-term cryopreservation did not substantially compromise this capacity, indicating phenotypic stability of the trait in environmental pathogens.

We used SEM to observe early-stage cultures (24 h) of these three bacteria, revealing significant morphological differences between the biofilm-producing and NB strains. The surfaces of the biofilm-producing bacteria exhibited a notable abundance of filamentous materials, leading to extensive aggregation and multidimensional stacking of the bacteria and their clonal strains. In contrast, the surfaces of the NB strains were relatively smooth and flat, with no observable filamentous material. Cell distribution appeared sparse and independent, with no signs of bacterial aggregation. On SEM, the filamentous fibers produced on the cell surface of *S. aureus* were shorter but had a higher density than those produced on the cell surface of *A. baumannii* and *P. aeruginosa*.

The crystal violet staining method remains the preferred approach for preliminary biofilm screening owing to its operational simplicity and broad applicability (18). We used this method to assess and compare the biofilm-forming capacities of three pathogens. Our findings validate its reliability as a standardized protocol for biofilm detection in clinical microbiology laboratories. The crystal violet staining method used here primarily evaluates the intrinsic potential of strains to form biofilms under optimized liquid conditions. Strains demonstrating strong biofilm formation in this standard model can be regarded as high-risk candidates for establishing persistent contamination on ICU surfaces. Future studies should validate the long-term survival and community formation of such strains under conditions that more closely mimic real-world dry-surface environments, including specific material types, humidity fluctuations, and nutrient limitation.

The three bacteria were sourced from the environment of healthcare ICUs, which are often key areas for environmental cleaning with high dosages and frequencies of disinfectant use. Thus, we hypothesize that under the strong selective pressure exerted by intensive environmental disinfection, strains capable of adhering to surfaces via biofilm formation, which enables them to resist both physical removal and chemical disinfection, may be selected for and persist in the ICU environment.

The preprocessing of the SEM specimens provided strong evidence for this hypothesis. After culturing the bacteria on cell crawls for 24 h, the specimens were washed three times with phosphate-buffered saline (PBS) before fixing with glutaraldehyde solution; the NB strains appeared sparse and dispersed in the microscope field, with no evidence of cell aggregation, suggesting that a significant number of bacteria were washed away with PBS, with only a few residual cells. In contrast, biofilm-producing strains showed substantial bacterial aggregation under the microscope, with even signs of multidimensional stacking. Although we only observed a subset of biofilm-forming strains using SEM, randomly selected strains obtained from the environmental surfaces of different hospitals exhibited highly consistent morphological characteristics. The surfaces of NB strains were relatively smooth and flat, with no observable filamentous materials, whereas SB strains consistently displayed similar surface filamentous fiber structures, indicating that these characteristics are common among strong biofilm-forming strains. Furthermore, the SEM observations are consistent with the findings of Flemming et al. (19), who reported that strong biofilm-forming strains commonly exhibit filamentous fiber structures on their surfaces, which play a crucial role in bacterial adhesion and biofilm formation. In this study, we utilized cell crawls, which have significantly smoother surfaces than materials used on patient beds and medical equipment surfaces in healthcare ICUs. The observed bacteria were only in the relatively early stage of biofilm formation and had not yet entered the biofilm maturation phase. These observations indicated that, unlike NB strains, biofilm-producing strains could firmly adhere to the glass surface and withstand three washes with PBS, resulting in visible cell aggregation. This may be related to the adhesive capabilities of the filamentous fibers on the cell surface. It is important to note that the FE-SEM images capture the architectural organization of cells at a fixed time point but cannot discriminate their metabolic states. While the observed dense aggregates and stacking are consistent with biofilm architecture, we cannot conclude whether the cells within these structures were actively growing, slow-growing, or in a dormant/persister state. Future studies employing viability staining, metabolic activity assays, or transcriptomic analysis on harvested biofilms would be required to address this important physiological question. If this hypothesis holds true, persistent microbial contamination in ICU environments that resists the physical decontamination effects of cleaning practices may not necessarily require bacterial biofilms to reach a mature stage—characterized by a dense 3D structure and abundant extracellular matrix (20). Instead, it can occur in the early stages of bacterial contamination as the filamentous fibers on the surfaces of biofilm-forming bacteria can tightly “grasp” environmental surfaces.

This study confirms that not all strains sourced from healthcare ICU environmental surfaces can produce biofilms. Nkemngong and Teska (21) observed that the differences between biofilm-forming and non-biofilm-forming strains lie in the bacterial surface proteins (such as protein A) and bacterial structures (such as pili) that enhance the adhesion of bacteria to abiotic surfaces. Although standardized culture conditions and screening techniques were implemented in this study, differences in biofilm-forming capabilities among the three bacteria were observed. In the case of *A. baumannii*, we identified four strains with varying biofilm-forming abilities: NB, WB, MB, and SB. This variation in capability was also evident in *P. aeruginosa* and *S. aureus*, strongly suggesting that biofilm formation is an inherent characteristic of these two strains. Further investigation is required to determine whether this ability is naturally acquired through environmental exposure.

The formation of bacterial biofilms is a complex, highly regulated, and multistage process (22) that involves inert substances and biological surfaces serving as substrates for the initial bacterial attachment, which is either reversible or irreversible (19). Through SEM observations, we could clearly observe differences in biofilm-formation capabilities among the strains. The NB strains exhibited a relatively smooth and flat cell surface, lacking filamentous fiber structures; thus, they were unable to withstand PBS washing. In contrast, the SB strains showed significant development of filamentous fibers on their surfaces. These fibers covered the bacteria and surfaces of various clonal strains, forming colonies of different sizes stacked in multiple dimensions. This structure promotes the tight adhesion of cells to various environmental surfaces, leading to irreversible attachment, which makes removal from the surface difficult (12). This also explains why target bacteria can still be recovered from environmental surfaces even after terminal disinfection measures have been implemented (9, 11). We propose that the filamentous fiber materials on bacterial surfaces, analogous to the role of “silk” in preparing a “scarf,” may initially play a crucial role in surface adhesion when bacteria come in contact with environmental surfaces. Subsequently, the “entrapment” effect of these fibers results in the accumulation of a large number of cells together. Furthermore, these fibers are likely to serve as foundational materials for bacterial biofilm formation.

Filamentous structures are crucial for promoting adhesion between cells and surfaces in the bacterial world (23). Furthermore, these fibers can serve as bridges for intercellular communication (24) and signaling mediators for biofilm formation, interconnecting cells located at different positions and intertwining to form a dense network or bundled structures that closely envelop or cover the cell surfaces, thereby establishing the initial framework of the biofilm. Various protein components within the biofilm matrix can aggregate into filamentous structures, providing structural rigidity and mechanical stability to the biofilm matrix, and serving as surface adhesins in different contexts (25). The phenomenon of biofilm-related proteins forming amyloid fibers was initially discovered in *Escherichia coli* (26), and similar amyloid fibers have also been observed in biofilms of other bacteria, such as FapC fibers in *P. aeruginosa* (27) and TasA fibers in *Bacillus subtilis* (28). Importantly, these amyloid fibers possess remarkable mechanical strength, self-assemble into filaments, and strongly promote cell-cell aggregation and surface adhesion. These properties closely match the characteristics of the dense, bundled, and multi-cell-connecting filaments observed in our study.

In healthcare settings, it is well established that the presence of DSBs increases the risk of microbial transmission (29) and poses substantial challenges to conventional environmental cleaning practices and surface disinfectants (30). In hospital settings, IPC should not be limited to changes in environmental cleaning strategies, regulations for environmental service personnel, or introduction of new disinfection technologies. Addressing microorganisms, with a focus on the relationship between DSBs and persistent environmental contamination, is also imperative. This necessitates comprehensive fundamental research into the mechanisms of bacterial biofilm formation, transmission, and their interactions with the environment to provide a scientific basis for sanitation in healthcare environments and patient medical safety.

This study has several limitations. First, since the samples were collected from only four tertiary teaching hospitals, the findings may not be fully generalizable to other regions or types of healthcare facilities. Second, the bacterial strains were isolated from environmental sources approximately a decade ago; therefore, we lack longitudinal data on the evolution of their biofilm-forming capacity. Additionally, the disinfectant tolerance of the biofilms was not assessed. Future studies should compare the resistance of strong and non-biofilm-forming strains to common hospital disinfectants with diverse mechanisms of action, including chlorine-based compounds, quaternary ammonium salts, and alcohols. A critical limitation of this descriptive study is that we were unable to biochemically characterize these key filamentous structures. Future work will be essential to determine their specific composition—for example, whether they represent specific pilin proteins, amyloid-forming proteins, or other surface polymers—by integrating proteomic analyses, specific enzymatic treatments, and gene-knockout approaches. Identifying their composition will not only help clarify the molecular basis of persistent contamination in healthcare settings but may also provide precise targets for developing enzyme-based cleaners or novel disinfection strategies designed to interfere with the initial bacterial adhesion process. However, we believe that this study has a broad relevance. The filamentous fiber structures observed on bacterial surfaces warrant further investigation in terms of their effect on bacterial adhesion to environmental surfaces and the mechanisms of biofilm formation.

This is the first study to demonstrate the biofilm-forming capacity and morphological characteristics of *A. baumannii*, *P. aeruginosa*, and *S. aureus* in ICU environments, filling a critical gap in our understanding of biofilm mechanisms among healthcare-associated pathogens. Notably, *P. aeruginosa* exhibited an 88.89% biofilm formation rate, with its distinctive filamentous fibrous structures significantly enhancing bacterial adhesion and aggregation—a key explanation for persistent environmental contamination. These findings directly inform the optimization of ICU cleaning protocols, promote the development of biofilm-targeted disinfection standards, and provide a scientific foundation for refining environmental monitoring metrics in infection control policies, ultimately reducing HAI rates.

## MATERIALS AND METHODS

### Strain acquisition and preservation

Routine environmental hygiene monitoring samples were collected between 2012 and 2015 from high-touch surfaces in six ICUs across four tertiary teaching hospitals (three in Hangzhou, Zhejiang Province and one in Nanjing, Jiangsu Province). High-touch surfaces included bed rails, bedside tables, monitor and control panels, computer keyboards at nursing stations, room door handles, and similar frequently contacted areas. A sterile 5 cm × 5 cm template was placed on the target surface. Using a sterile cotton swab pre-moistened with 0.03 mol/L PBS or physiological saline, we swabbed the template area systematically with five horizontal and five vertical strokes while rotating the swab. Sampling extended to one to four contiguous template areas when necessary. The swab handle was broken off, and the tip was transferred to a sterile tube containing 10 mL of sampling solution for analysis. For small surfaces (e.g., door handles), the entire area was swabbed directly. If residual disinfectants were suspected, neutralizing agents were added to the sampling solution. After vortex-mixing the solution vigorously, 1.0 mL aliquots from serial dilutions were plated on agar plates. Each plate was overlaid with 15–20 mL of molten nutrient agar (cooled to 40–45°C), solidified, and incubated at 36 ± 1°C for 48 h. Bacterial colonies were counted; the results are expressed as colony-forming units per surface area. The isolates were identified using the matrix-assisted laser desorption ionization-time of flight mass spectrometer, according to manufacturer’s instructions (VITEK MS; bioMérieux Shanghai Co., Ltd., Shanghai, China). The strains were preserved at −80°C using ceramic bead technology. For revival, the ceramic beads were picked from the stock tube and transferred onto a blood agar plate several times. Thereafter, the plates were incubated at 36°C for 48 h. Successfully revived strains were subjected to continuous passaging, and the third generation was selected for subsequent experiments.

### Biofilm formation assay

Each isolated strain was inoculated in tryptic soy broth medium. When the bacteria reached the logarithmic growth phase (OD_600_ = 0.4–0.6), the bacterial suspension was transferred to a 96-well plate. Biofilm formation was assessed using the crystal violet staining method according to Lou et al. (31). Each strain was tested in six replicate wells and incubated at 37°C for 24 h. Wells containing only sterile TSB served as the negative controls. The optical density (OD) of each well was measured at 570 nm. The cutoff value (ODC) was defined as the mean OD of the negative control wells plus three times the standard deviation (3 × SD). Based on the OD/ODC ratio, strains were categorized as follows: NB strains (OD/ODC ≤ 1), WB strains (1 < OD/ODC ≤ 2), MB strains (2 < OD/ODC ≤ 4), and SB strains (OD/ODC > 4).

### FE-SEM

Strains with different biofilm-forming abilities were inoculated onto microtiter plates containing cell culture slides. Following a 24-h incubation, the medium was removed and the slides were gently washed with buffer to remove planktonic bacteria. Subsequently, the samples were fixed in 2.5% glutaraldehyde solution overnight at 4°C, rinsed thrice with 0.1 M PBS (15 min each), post-fixed with 1% osmium tetroxide for 1.5 h, and rinsed again with PBS. Dehydration was performed using an ethanol series (30%, 50%, 70%, 90%, 95%, and 100%; 15 min per concentration). After critical point drying, the samples were subjected to gold sputter coating and examined using FE-SEM (Nova Nano 450; Thermo FEI, Hillsboro, OR, USA) at magnifications of 3,500×, 12,000×, and 35,000× to examine the morphological characteristics of the cell surfaces. For each strain examined, FE-SEM observation was conducted systematically across at least three random and non-contiguous fields of view to ensure an unbiased assessment. Images at three magnifications were captured for each field. The micrographs presented in the figures were selected as the most representative images that clearly illustrated the morphological features consistently observed across the majority of fields for that particular biofilm-forming category.

### Data analysis

Categorical data, such as the distribution of biofilm-forming abilities, were presented as frequencies and percentages. Differences in the distribution of biofilm-forming capabilities (NB, WB, MB, and SB) among the three bacterial species were assessed using Pearson’s chi-square test. If the overall chi-square test result was significant (*P* < 0.05), post-hoc pairwise chi-square tests were conducted, and the significance level was adjusted using the Bonferroni correction (adjusted *α*′ = 0.017) to account for multiple comparisons. All statistical analyses were performed using GraphPad.

## Data Availability

The data set can be accessed via the following DOI: https://doi.org/10.5281/zenodo.18414738.

## SUPPLEMENTAL MATERIAL

Fig. S1 spectrum.02114-25-s0001.tiff Biofilm-forming capacity of *Acinetobacter baumannii* isolates.

Fig. S2 spectrum.02114-25-s0002.tiff Biofilm-forming capacity of *Pseudomonas aeruginosa* isolates.

Fig. S3 spectrum.02114-25-s0003.tiff Biofilm-forming capacity of *Staphylococcus aureus* isolates.

Supplemental legends spectrum.02114-25-s0004.doc Descriptive legends for Fig. S1 to S3.

ASM does not own the copyrights to Supplemental Material that may be linked to, or accessed through, an article. The authors have granted ASM a non-exclusive, world-wide license to publish the Supplemental Material files. Please contact the corresponding author directly for reuse.
