# Supplementary figures and images for: Filamentous surface structures drive biofilm formation in ICU-isolated Acinetobacter baumannii, Pseudomonas aeruginosa, and Staphylococcus aureus: implications for persistent environmental contamination
Source: Microbiol Spectr. 2026 Mar 13;14(4):e02114-25. doi: 10.1128/spectrum.02114-25 (PMC13055353; doi:10.1128/spectrum.02114-25)

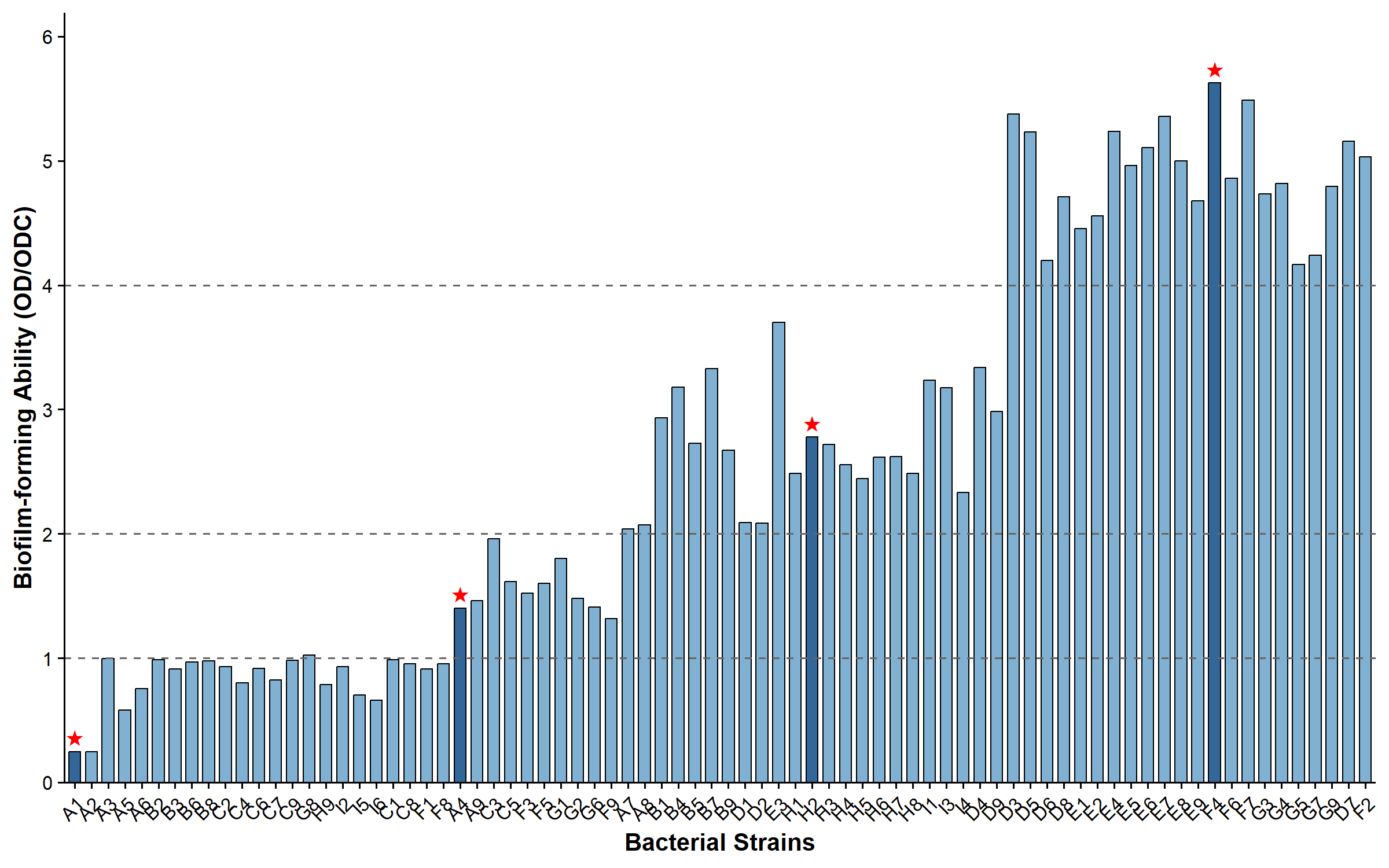

Supplement: Fig. S1 — Biofilm-forming capacity of Acinetobacter baumannii isolates. [file spectrum.02114-25-s0001.tiff]

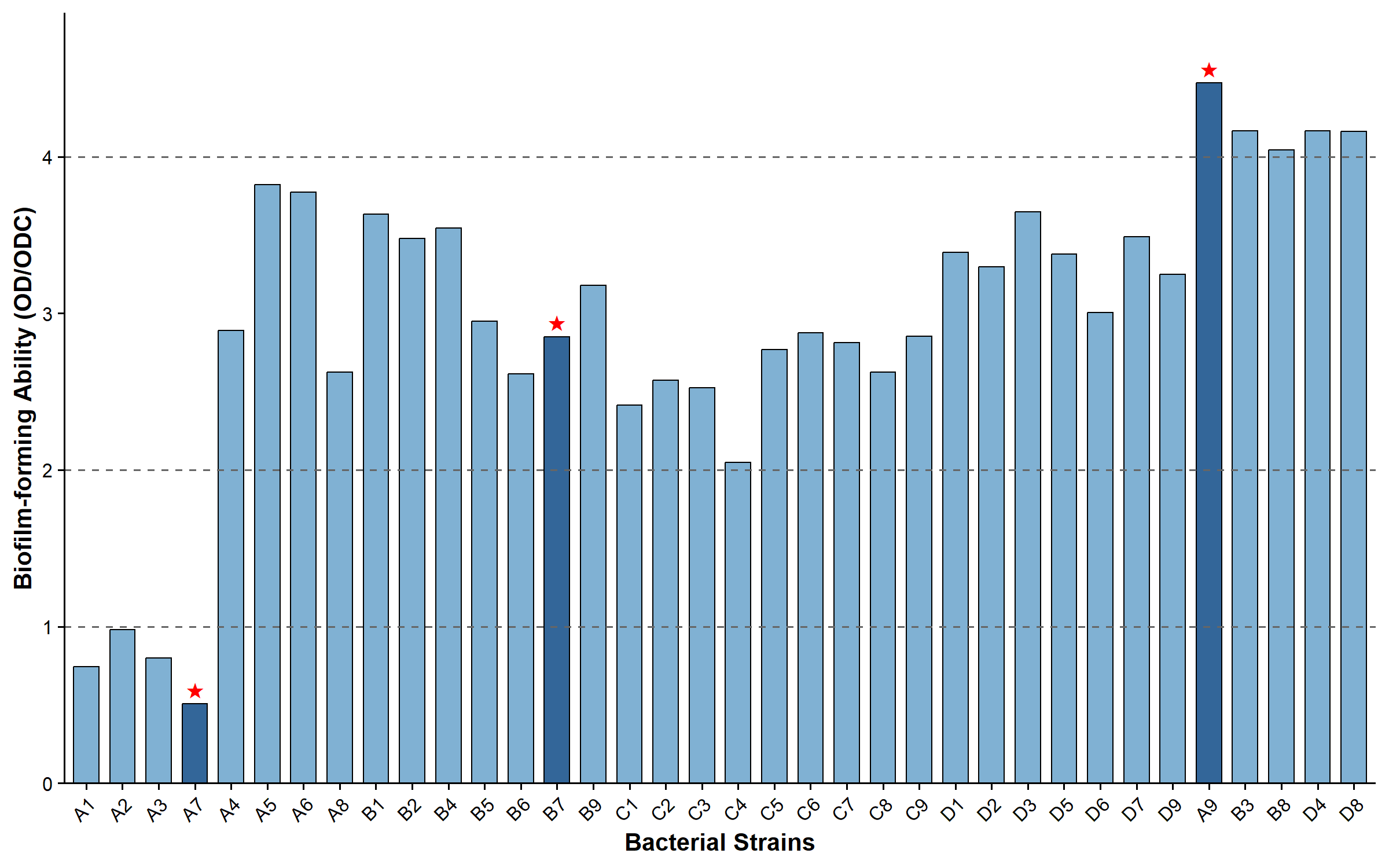

Supplement: Fig. S2 — Biofilm-forming capacity of Pseudomonas aeruginosa isolates. [file spectrum.02114-25-s0002.tiff]

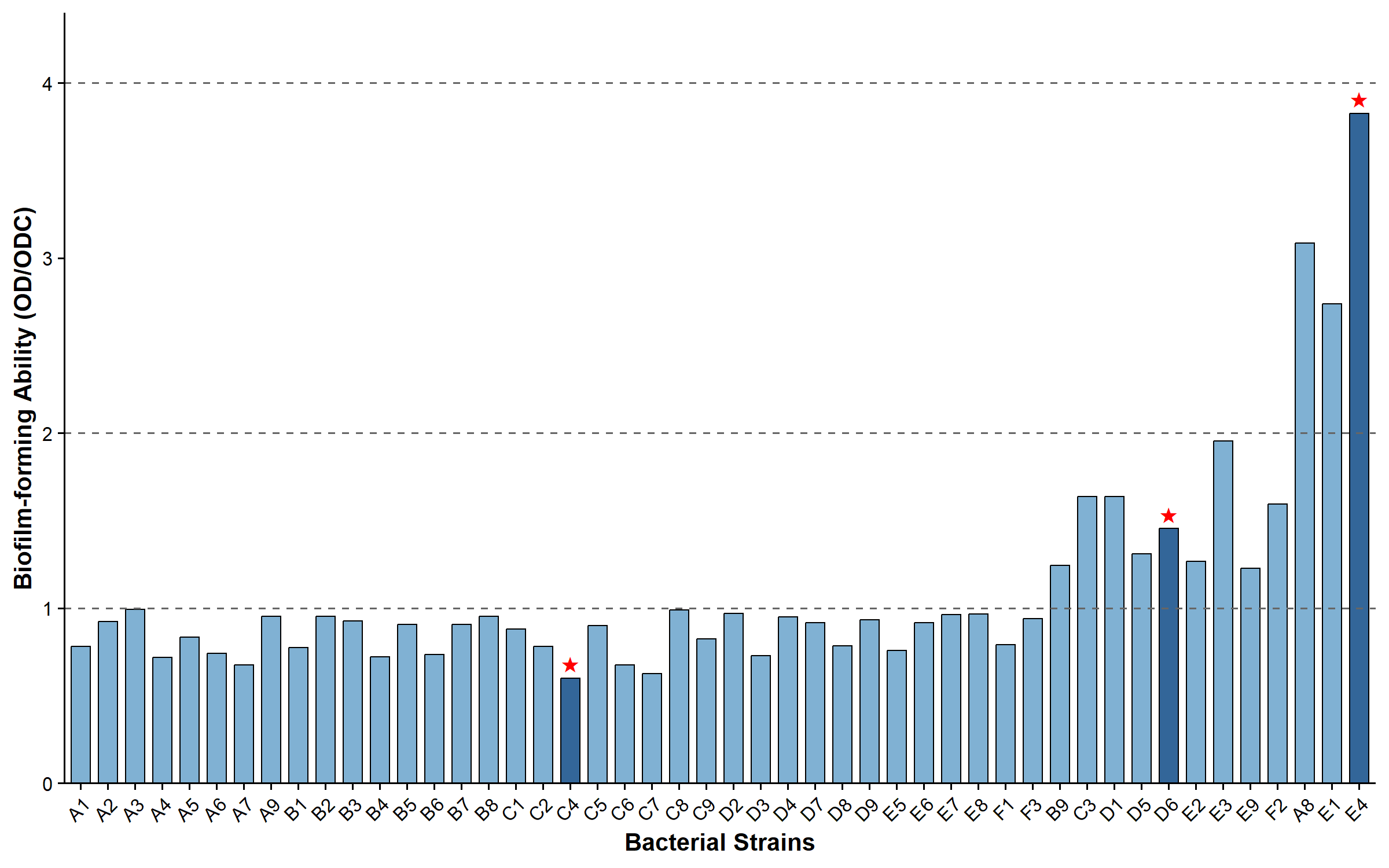

Supplement: Fig. S3 — Biofilm-forming capacity of Staphylococcus aureus isolates. [file spectrum.02114-25-s0003.tiff]
