# Supplementary material for: Filamentous surface structures drive biofilm formation in ICU-isolated Acinetobacter baumannii, Pseudomonas aeruginosa, and Staphylococcus aureus: implications for persistent environmental contamination
Source: Microbiol Spectr. 2026 Mar 13;14(4):e02114-25. doi: 10.1128/spectrum.02114-25 (PMC13055353; doi:10.1128/spectrum.02114-25)
Supplement: Supplemental legends — Descriptive legends for Fig. S1 to S3. [file spectrum.02114-25-s0004.doc]

**Supplementary Figure Legends**

**Supplementary Figure S1:** Biofilm-forming capacity of *Acinetobacter baumannii*isolates. Bar graphs display the raw crystal violet absorbance (OD570) for each individual strain tested. The dashed horizontal lines represent the optical density cutoff (ODC) values used to categorize strains as: non-biofilm forming (NB, OD/ODC ≤ 1), weak (WB, 1 < OD/ODC ≤ 2), moderate (MB, 2 < OD/ODC ≤ 4), or strong (SB, OD/ODC > 4) biofilm formers. Strains selected for scanning electron microscopy (SEM) analysis are explicitly labeled with their identifiers.

**Supplementary Figure S2:** Biofilm-forming capacity of *Pseudomonas aeruginosa* isolates. Bar graphs display the raw crystal violet absorbance (OD570) for each individual strain tested. The dashed horizontal lines represent the optical density cutoff (ODC) values used to categorize strains as: non-biofilm forming (NB, OD/ODC ≤ 1), weak (WB, 1 < OD/ODC ≤ 2), moderate (MB, 2 < OD/ODC ≤ 4), or strong (SB, OD/ODC > 4) biofilm formers. Strains selected for scanning electron microscopy (SEM) analysis are explicitly labeled with their identifiers.

**Supplementary Figure S3:** Biofilm-forming capacity of *Staphylococcus aureus* isolates. Bar graphs display the raw crystal violet absorbance (OD570) for each individual strain tested. The dashed horizontal lines represent the optical density cutoff (ODC) values used to categorize strains as: non-biofilm forming (NB, OD/ODC ≤ 1), weak (WB, 1 < OD/ODC ≤ 2), moderate (MB, 2 < OD/ODC ≤ 4), or strong (SB, OD/ODC > 4) biofilm formers. Strains selected for scanning electron microscopy (SEM) analysis are explicitly labeled with their identifiers.
